# A 55-year-old man with mild shortness of breath

**DOI:** 10.1007/s12471-019-01313-z

**Published:** 2019-07-23

**Authors:** A. D. Egorova, J. M. Smit, P. Kiès

**Affiliations:** Department of Cardiology, Heart Lung Centre, Leiden University Medical Centre, Leiden, The Netherlands

## Answer

The correct answer is C.

**Fig. 1:**
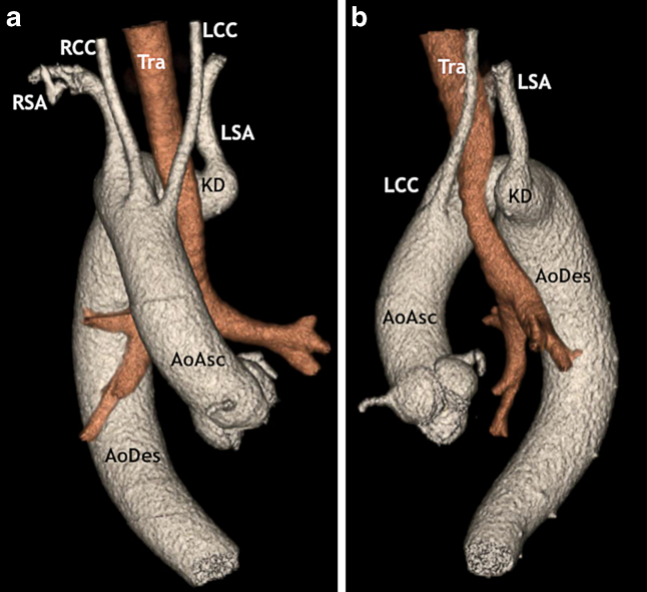
A three-dimensional reconstruction of the CT angiography showing the frontal view (**a**) and the left lateral view (**b**) of the right-sided aortic arch giving rise to the aberrant left subclavian artery from the Kommerell diverticulum. (*AoAsc* ascending aorta, *AoDes* descending aorta, *Tra* trachea, *LCC* left common carotid artery, *RCC* right common carotid artery, *RSA* right subclavian artery, *LSA* aberrant left subclavian artery, *KD* Kommerell diverticulum)

The chest radiograph shown in Fig. 1a of the Question shows the silhouette of a right-sided aortic arch coursing to the right of the trachea. This is a rare, often asymptomatic and incidental finding in the setting of an otherwise structurally normal heart, which was first described in 1763 [1]. It is estimated to occur in 0.04–0.1% of the population. About half of the cases are associated with an aberrant left subclavian artery, as is illustrated in our patient (type II right-sided aortic arch variant). Other types of right-sided aortic arch variants include mirror-image arch branches (type I) and an isolated left subclavian artery communicating with the pulmonary artery (type III). Type I right aortic arch in particular is strongly associated with congenital heart disease, most commonly tetralogy of Fallot.

There is no evidence of acute aortic pathology (associated with a second ‘false’ lumen and dissection flap in the aortic wall), left persistent vena cava superior (associated with a dilated coronary sinus) or a double aortic arch on the images presented. Therefore, we can exclude these options.

A three-dimensional reconstruction of the CT scan (Fig. 1a, b) facilitates our comprehension of the anatomy of the right-sided aortic arch giving rise to a left and right common carotid artery, a right subclavian artery and an aberrant left subclavian artery. The left subclavian artery originates from a vascular pouch, referred to as the Kommerell diverticulum (alternatively called remnant diverticulum or lusoria root) posterior to the trachea and oesophagus, potentially compressing these structures. Its aneurysmatic enlargement can lead to dissection and rupture, making it an important clinical phenomenon to recognise [2]. CT and MRI are essential tools for evaluation of this pathology. The timing of the (often complex surgical) management of this congenital pathology remains a matter of debate.
